# Artificial neural networks as a prognostic tool using hyperspectral imaging on pretherapeutic histopathological specimens of esophageal adenocarcinoma

**DOI:** 10.1007/s00432-025-06340-5

**Published:** 2025-10-04

**Authors:** Christel Teresa Trifone, Marianne Maktabi, Philipp Bischoff, Katrin Schierle, Stefan Niebisch, Yusef Moulla, Patrick Sven Plum, Boris Jansen-Winkeln, Ines Gockel, René Thieme

**Affiliations:** 1 Department for General and Abdominal Surgery, Hospital Chemnitz gGmbH, Chemnitz, Germany; 2 Innovation Center Computer Assisted Surgery (ICCAS), University of Leipzig, LeipzigLeipzig, Germany; 3 Department of Electrical, Mechanical and Industrial Engineering Köthen, University of Applied Science Anhalt, Bernburg, Germany; 4 Institute of Pathology, SLK-Kliniken Heilbronn, Heilbronn, Germany; 5 Department of Visceral, Transplant, Thorax and Vascular Surgery, University Hospital of Leipzig, Leipzig, Germany; 6 Department of General, Visceral, Thoracic and Vascular Surgery, St. George’s Hospital Leipzig, Leipzig, Germany; 7 Department of Visceral, Vascular and Emergency Surgery, Klinikum Magdeburg gGmbH, Magdeburg, Germany

**Keywords:** Esophageal adenocarcinoma, Hyperspectral imaging (HSI), Therapy prediction, Digital pathology, Personalized treatment

## Abstract

**Purpose:**

The integration of artificial intelligence (AI) with hyperspectral imaging (HSI) offers a promising avenue for improving pre-therapeutic prognosis, a key factor in optimizing cancer treatment strategies. This study explores the potential of artificial neural networks (ANNs) to predict the effectiveness of preoperative chemo- or radiochemotherapy in esophageal adenocarcinoma (EAC), using HSI data derived from histopathological tissue samples.

**Methods:**

HSI data were obtained from pre-therapeutic histopathological samples of 21 patients with EAC. Following annotation and spectral extraction, the data underwent pre-processing steps including normalization, shuffling, and batch organization. Three artificial neural network (ANN) models—2D convolutional neural networks (2D-CNNs), 3D convolutional neural networks (3D-CNNs), and Hybrid-Spectral Networks (Hybrid-SN)—were trained to predict treatment response. Model performance was assessed using sensitivity, specificity, accuracy, and F1-score, offering insights into their clinical utility

**Results:**

The 3D-CNN model achieved the highest accuracy (0.68 ± 0.09) and F1-score (0.66 ± 0.08), highlighting its strength in capturing both spatial and spectral information. The Hybrid-SN model demonstrated the highest sensitivity (0.79 ± 0.19), indicating strong performance in identifying responders to neoadjuvant therapy. In contrast, the 2D-CNN model achieved the highest specificity (0.73 ± 0.15), reflecting its effectiveness in correctly identifying non-responders.

**Conclusion:**

This study demonstrates the potential of combining HSI with ANNs to predict treatment response in EAC. Among the models evaluated, the 3D-CNN showed the most balanced performance, effectively leveraging spatial and spectral features, while the Hybrid-SN and 2D-CNN models excelled in sensitivity and specificity, respectively. These findings underline the feasibility of using AI-driven analysis of histopathological HSI data to support personalized treatment planning in EAC, paving the way for more accurate and tailored therapeutic strategies.

## Introduction

Globally, esophageal cancer accounted for approximately 604,100 new cases and 544,100 deaths in 2022, according to GLOBOCAN estimates (Bray et al. 2024). Esophageal squamous cell carcinoma (ESCC) and esophageal adenocarcinoma (EAC) are the most frequent histological types. ESCC accounts for most cases globally (85%), while EAC represents 14% of the total burden, with significant variation in subtype distribution by region and risk factors (Morgan et al. 2022). EAC is a considerable health concern due to its aggressive nature, rising incidence, and high mortality rates, particularly in Western countries, where its incidence has increased during the last decades (Souza and Spechler 2022). The disease predominantly affects males and is closely linked to risk factors including obesity, gastroesophageal reflux disease (GERD), and tobacco use (Souza and Spechler 2022). Barrett’s esophagus, characterized by the replacement of normal squamous mucosa with metaplastic columnar epithelium in the distal esophagus, is regarded as the primary precursor to EAC. This premalignant condition develops because of chronic GERD, which damages the esophageal lining and triggers molecular alterations in cells at the esophagogastric junction (Souza and Spechler 2022). The rising incidence of EAC emphasizes the need for effective early detection strategies and the development of prognostic tools to enhance patient outcomes and enable personalized treatment approaches. Esophagogastroduodenoscopy (EGD) serves as the primary diagnostic procedure for suspected esophageal cancer, during which biopsies of abnormal lesions are obtained.

Treatment decisions for esophageal adenocarcinoma (EAC) are guided by cancer stage and individual patient and clearly defined prognostic and risk factors. Therapeutic approaches have advanced considerably, with multimodal neoadjuvant strategies employed when primary resection is not indicated. Preoperative chemotherapy, radiochemotherapy, and immunotherapy are commonly used to downstage tumors prior to surgery. The FLOT4-AIO trial demonstrated that perioperative FLOT chemotherapy significantly enhances both complete pathological remission rates and overall survival in patients with resectable gastric and gastroesophageal adenocarcinoma, achieving a three-year survival rate of 57% (Al-Batran et al. 2016, 2019). A prospective-randomized comparison between perioperative FLOT chemotherapy and neoadjuvant radiochemotherapy using the CROSS protocol revealed that FLOT significantly improved progression-free survival, with rates of 51.6% versus 35.0%, respectively (Hoeppner et al. 2025). Immune checkpoint inhibitors have shown efficacy in the treatment of EAC, with the addition of monoclonal antibodies, such as Nivolumab or Pembrolizumab, to chemotherapy resulting in improved overall survival (Janjigian et al. 2021; Sun et al. 2021).

Accurately predicting tumor regression in response to therapy is essential for effective treatment planning and improved outcomes in EAC. Current prediction models aim to estimate the likelihood of response to neoadjuvant therapy. These include genetic markers, such as alterations in the TP53 pathway, which are linked to poor therapeutic response, as well as histology-based deep learning models that leverage morphological tissue biomarkers to make highly accurate predictions (Sihag et al. 2022; Hörst et al. 2023).

The integration of artificial intelligence (AI) into clinical workflows supports the development of advanced assistive technologies. Combining AI with HSI has already demonstrated promising potential in tumor tissue classification (Knospe et al. 2022; Maktabi et al. 2022; Pathak et al. 2023). HSI is an imaging technique that records a broad spectrum of light across multiple wavelengths for each pixel, producing a rich, three-dimensional dataset, known as a hypercube, which contains both spatial and spectral information (Lu and Fei 2014). HSI enables the differentiation of materials based on their unique spectral signatures, offering insights into the compositional, physiological, and morphological properties of tissues (Calin et al. 2014). These capabilities make HSI a valuable asset in medical applications, especially in surgical and pathological contexts.

HSI enables real-time, non-invasive tissue characterization during surgery and is increasingly used in pathology for the automated detection and classification of cancerous tissues, including EAC (Maktabi et al. 2022), breast cancer (Jong et al. 2022), pancreatic cancer (Ishikawa et al. 2019), thyroid cancer (Tran et al. 2024), and gastric cancer (Du et al. 2023). When combined with machine learning and deep learning algorithms, HSI’s diagnostic performance is further enhanced. For instance, one study showed that integrating HSI with a neural network could distinguish colorectal cancer from healthy mucosa with 86% sensitivity and 95% specificity (Jansen-Winkeln et al. 2021). In parallel, deep learning approaches applied to digitized histological slides have shown strong potential for predicting tumor characteristics. A retrospective study demonstrated that AI significantly improved diagnostic accuracy and reduced assessment time in the evaluation of EAC histological specimens, highlighting its value in modern pathology (Tolkach et al. 2023).

Despite significant advancements, a critical gap remains in the ability to predict treatment success in EAC. This study addresses that need by exploring the integration of HSI with advanced artificial neural network (ANN) architectures to forecast the effectiveness of neoadjuvant therapy. Accurate early prediction of therapeutic outcomes is essential for guiding personalized treatment strategies and improving patient prognosis. By capturing rich spectral data from histopathological specimens, HSI provides a valuable foundation for ANN-based analysis. This research evaluates the predictive potential of HSI in combination with three ANN models, 2D convolutional neural networks (2D-CNN), 3D convolutional neural networks (3D-CNN), and Hybrid-Spectral Networks (Hybrid-SN), for assessing neoadjuvant therapy response in EAC patients.

## Methods

### Study design and patient selection

This retrospective study was conducted at the University Hospital of Leipzig (UKL, Germany) using histopathological sections from patients diagnosed with EAC between January 2014 and March 2020. Eligible patients were those diagnosed with EAC at the UKL, who received neoadjuvant chemo- or radiochemotherapy followed by surgical resection (esophagectomy) at the same institution. The study cohort consisted of 21 patients, with hematoxylin and eosin (HE)-stained biopsy samples collected under standardized conditions. All histological sections were provided by the Institute of Pathology at the University Hospital of Leipzig. The study was conducted in accordance with the Declaration of Helsinki and approved by the local Ethics Committee of the Medical Faculty of the University of Leipzig (approval number 307-15).

Patients received different neoadjuvant treatment regimens, including FLOT (5-fluorouracil, leucovorin, oxaliplatin, and docetaxel), FLO (5-fluorouracil, leucovorin, and oxaliplatin), the CROSS protocol, e.g., in the context ot the ESOPEC study (chemoradiotherapy with carboplatin and paclitaxel, plus 41.4 Gy radiation), and the INNOVATION study regimen (FLOT combined with targeted therapy using Trastuzumab and Pertuzumab). Following neoadjuvant therapy, patients underwent either a single-stage esophagectomy or a two-stage procedure, encompassing laparoscopic gastrolysis with ischemic conditioning, followed by gastric tube formation, esophagectomy, and gastric pull-up with intrathoracic anastomosis, about 3–7 days later.

For inclusion in this retrospective study, only cases with complete histopathological analysis following resection were considered. To minimize potential bias, only patients for whom both pre-therapeutic and post-operative histopathological sections were assessed, were included, ensuring consistency in examination and interpretation.

Initially, 109 patients were screened for potential inclusion. After applying the defined inclusion and exclusion criteria, a final cohort of 21 patients remained. The most common reason for exclusion was the unavailability of histopathological slides from the initial diagnosis of EAC (pre-therapeutic), as they had been taken and diagnosed outside the University Hospital of Leizpig.

### HSI recording

Following patient selection and retrieval of HE-stained slides, an experienced gastrointestinal pathologist (KS) annotated tumor cell regions in the pre-therapeutic samples to confirm the presence of malignancy. In the postoperative specimens, areas representing varying degrees of tumor regression, were identified to support accurate histopathological characterization. The cohort encompassed all four histopathological tumor regression stages, ranging from complete to minimal response to neoadjuvant therapy. Histopathological evaluation included assessment of tumor regression using the ypTNM-classification and tumor regression grading systems proposed by Werner and Höfler (Werner and Höfler 2000) and Schneider et al. (Schneider et al. 2005). These systems quantify tumor response to neoadjuvant therapy based on residual tumor cellularity, necrosis, and fibrosis, thereby serving as indicators of treatment efficacy and potential guides for subsequent clinical management.

Selected HE slides were digitalized for virtual microscopy. Prior to imaging, all previous annotations were carefully removed. Hyperspectral imaging (HSI) was then performed using an HSI camera system (Diaspective Vision GmbH, Am Salzhaff-Peplow, Germany) mounted on an AxioVision microscope (Carl Zeiss Microscopy GmbH, Jena, Germany). The system captured images across a spectral range of 500 nm to 745 nm, using a 20 × objective and an integrated LED light source (Carl Zeiss Microscopy GmbH) as described previously (Maktabi et al. 2022).

Representative images of both healthy and malignant cell clusters were acquired from pre-therapeutic and postoperative HE slides. Healthy tissue was identified as squamous epithelium, whereas malignant areas were classified as adenocarcinoma, either at initial diagnosis or at the time of post-treatment, capturing various stages of tumor regression.

Tumor regions on the digitalized slides were annotated using ZEISS ZEN 3.3 (blue edition) software (Carl Zeiss Microscopy GmbH, Jena, Germany), allowing for precise definition of regions of interest (ROI). Annotation was supervised by the GI pathologist (KS) to ensure accurate tumor boundary delineation. Only pathological areas were marked with a black outline and fill; healthy tissue and background were deliberately left unannotated. Both pre-treatment and post-surgical samples were included to encompass the full spectrum of tumor regression. These detailed annotations formed the foundation for subsequent data analysis and were essential for training artificial neural networks. Based on the regression grading observed in the postoperative histopathological specimens, cases were classified into the “Responder” or the “Non-Responder” group.

### Data extraction, preprocessing, and feature extraction

A total of 32 hyperspectral data cubes were acquired and stored as multidimensional arrays, capturing high-resolution spectral information for each pixel across wavelengths from 500 to 745 nm. These data cubes included samples from both healthy and tumor tissues, with some patients contributing data for both types. Care was taken to avoid data duplication, and each patient was represented by one to three hyperspectral data cubes.

Prior to analysis, the data underwent several pre-processing steps. First, z-score normalization was applied to standardize the spectral data, adjusting each feature to have a mean of 0 and a standard deviation of 1. The z-score normalization is a well-known method for normalization hyperspectral imaging data (Tkachenko, Maktabi). The normalization was followed by random shuffling of the dataset to reduce sampling bias during model training. No further smoothing was applied to the data.

### Model selection, architecture, and hyperparameter tuning

**Fig. 1:**
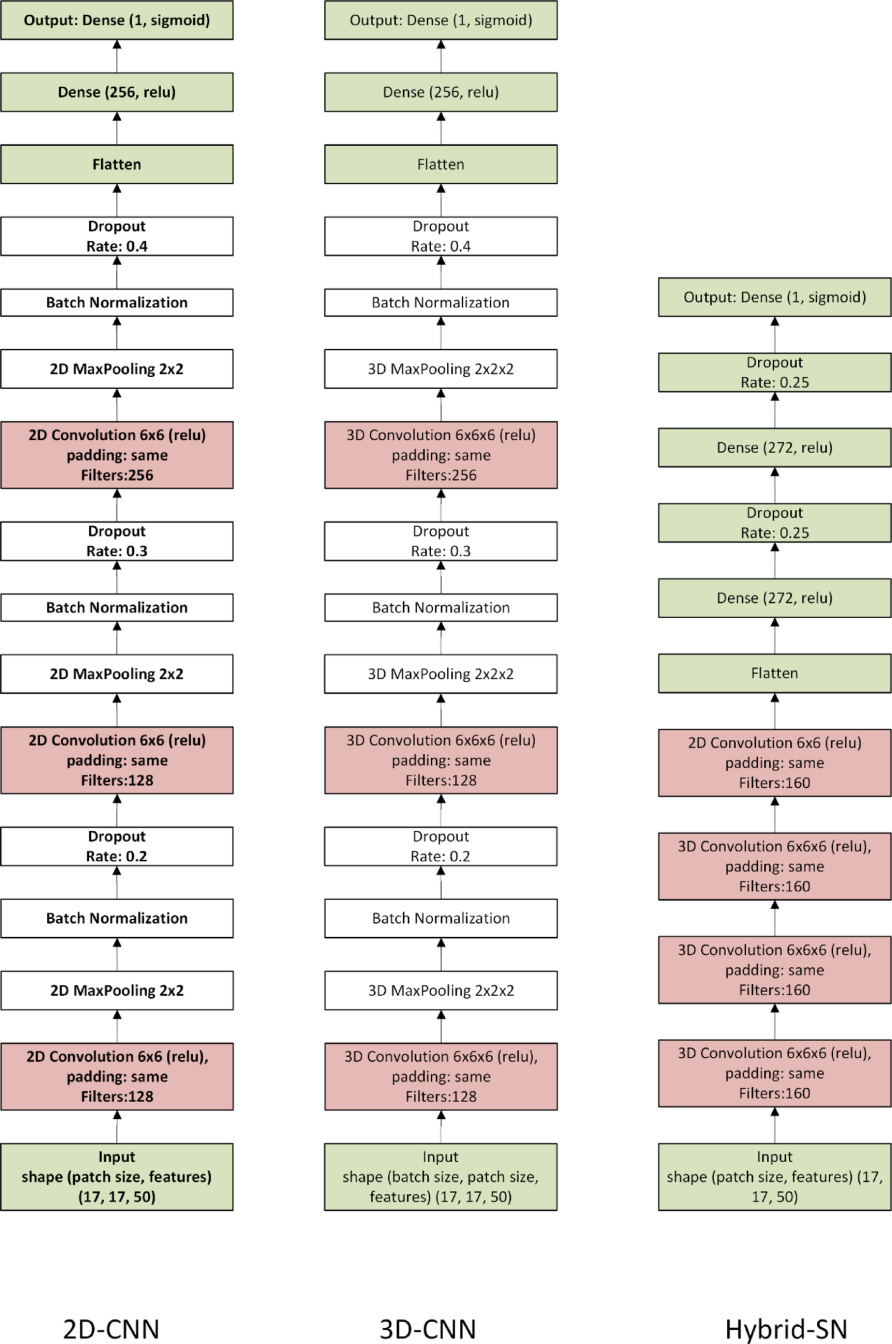
Architectures that were used: 2D-CNN (left), 3D-CNN (middle) and Hybrid-SN (right)

To predict patient prognosis, three neural network architectures were employed: a 2D Convolutional Neural Network (2D-CNN), a 3D Convolutional Neural Network (3D-CNN), and the Hybrid Spectral Network (Hybrid-SN), as proposed by Ben Hamida et al. (Ben Hamida et al. 2018). These models were chosen for their effectiveness in processing hyperspectral imaging (HSI) data, leveraging both spatial and spectral information. The 2D-CNN model treated hyperspectral data as a series of 2D image patches, each corresponding to a single spectral band. Spatial features were extracted independently from each band using 2D convolutional layers, followed by pooling layers to reduce dimensionality while preserving essential features. The architecture comprised three convolutional layers with 128 and 256 filters, followed by Max Pooling and Dropout layers to prevent overfitting. The final layers included a Flatten operation and two Dense (fully connected) layers with 256 and 1 neuron(s), respectively. The 3D-CNN model processed hyperspectral data as volumetric input, enabling the simultaneous extraction of spatial and spectral features through 3D convolutional filters. This architecture was particularly suited for capturing complex inter-band relationships. Similar to the 2D-CNN, the 3D-CNN included three convolutional layers with 128 and 256 filters, followed by Max Pooling, Dropout, Flatten, and two Dense layers (256 and 1 neuron). The Hybrid Spectral Network (Hybrid-SN) combined the strengths of both 2D and 3D CNNs. It began with 2D convolutional layers to extract spatial features from individual spectral bands and followed with 3D convolutional layers to integrate spectral and spatial information. This hybrid design enhanced the model's capacity to detect subtle, complex patterns across the hyperspectral data cube. Model performance was optimized through hyperparameter tuning using Bayesian optimization with 4000 trials. Parameters such as convolutional filter sizes (e.g., 6 × 6 for both 2D- and 3D-CNN), dropout rates, and the number of neurons in fully connected layers, were systematically adjusted. For the Hybrid-SN, the network depth and neuron count were specifically tailored to maximize predictive accuracy (Fig. 1).

### Training and evaluation metrics

Model training was conducted in a high-performance computing environment equipped with NVIDIA GPUs. The implementation utilized Python (version 3.7.4, Python Software Foundation, Wilmington, DE, USA), TensorFlow (version 2.4.0), and Keras (version 2.3). Training was performed on the University of Leipzig’s HPC cluster, featuring an AMD EPYC 32-core processor and two NVIDIA RTX 2080 Ti GPUs. Models were trained for 50 to 100 epochs with a batch size of 32 and a fixed learning rate of 0.0001. The Adam optimizer was used with hyperparameters β₁ = 0.9 and β₂ = 0.99. To prevent overfitting and enhance training efficiency, early stopping and learning rate scheduling were applied. As class-balancing strategy class weights were used based on calculating the quotient of the total number of samples and the number of samples of the specific class. A comprehensive description of the methods is provided in (Tkachenko et al. 2023). For evaluation, the hyperspectral dataset was divided on a per-patient basis into three groups for threefold cross-validation. Each fold included approximately two-thirds of the data for training and one-third for validation, ensuring that data from individual patients did not appear in both sets simultaneously. This cross-validation approach provided a robust estimate of model performance and generalizability. In threefold cross-validation, the dataset was split into three equal parts, and the model was trained and validated three times—each time using a different subset for validation, while the remaining two subsets are used for training. This method ensured that each sample contributed to both training and validation, resulting in a more reliable assessment of model accuracy. Three Patient data sets were used for validation and three patient data sets were used for testing. Hence, two thirds of the whole data were used to train the models. Model performance was evaluated using key metrics (Tkachenko et al. 2023):

- Accuracy: The proportion of true results among the total cases examined.
- Sensitivity (Recall): The model's ability to correctly identify positive cases.
- Specificity: The model's ability to correctly identify negative cases.
- F1-Score: The harmonic mean of precision and recall, balancing the two metrics.

## Results

### Study population

The study cohort consisted of 21 patients, with a mean age of 72.3 years (range 59–82 years), predominantly male (n = 20) and one female. Clinical and pathological characteristics, including demographic information, pretherapeutic tumor staging, neoadjuvant treatment protocols, surgical approaches, and postoperative pathological staging, are presented in detail in Table 1. Tumor regression grades for the cohort are summarized in Table 2, categorized as follows: Grade 1a (complete regression), Grade 1b (subtotal regression), Grade 2 (partial regression), and Grade 3 (minimal or no regression).

**Table 1:** Details of the cohort

Characteristics	Number of cases
Sample size	21
Mean age; range (years)	72.3 no,(59–82)
Gender	
Male	20
Female	1
cT-classification^a^—pretherapeutic	
cT1	1
cT2	1
cT3	14
cT4	0
cTX	3
n.a.^g^	2
cN-classification^b^—pretherapeutic	
cN0	3
cN+	13
cNX	3
n.a.^g^	2
cM-classification^c^—pretherapeutic	
cM0	12
cM+	2
cMX	1
n.a.^g^	6
Grading^d^—pretherapetic	
G1	2
G2	8
G3	7
n.a.^g^	4
Neoadjuvant/perioperative treatment	
CTx^e^ (FLO, FLOT, INNOVATION)	15 (1,12, 2)
RCTx^f^ (CROSS)	6
Type of surgical procedure	
Single-stage esophagectomy	6
Two-stage procedure (laparoscopic gastrolysis followed by time-shifted esophagectomy and gastric pull-up)	15
ypT-classification – postoperative	
ypT0	5
ypT1	1
ypT2	5
ypT3	10
ypT4	0
ypN-classification—postoperative	
ypN0	15
ypN1	3
ypN2	3

This table summarizes the clinical and pathological characteristics of the cohort, including demographic data, pretherapeutic tumor staging, neoadjuvant / perioperative treatment regimens, surgical approaches, and postoperative pathological staging

^a^T-classification = Tumor stage based on TNM classification

^b^N-classification = Nodal stage based on TNM classification

^c^M-classification = Distant metastasis based on TNM classification

^d^Grading = Histological tumor grading (G1 = well-differentiated, G2 = moderately differentiated, G3 = poorly differentiated, G4?)

^e^CTx = Chemotherapy (used regimens: FLO = fluorouracil, leucovorin, oxaliplatin; FLOT = fluorouracil, leucovorin, oxaliplatin, docetaxel; INNOVATION-study = targeted therapy combined with chemotherapy)

^F^RCTx = Radiochemotherapy (used regimen: CROSS protocol = carboplatin and paclitaxel with concurrent radiotherapy)

g: n.a. = Not available or missing data

**Table 2:** Regression gradings of the cohort according to Werner and Höfler (Werner and Höfler 2000)

Regression grading	Number of cases
TU Munich regression grading according to Werner and Höfler (2000)	
Grade 1a—complete response^a^	5
Grade 1b—subtotal response^b^	3
Grade 2—partial response^c^	10
Grade 3—minimal/no response^d^	3

This table summarizes the histopathological tumor regression grades observed in the cohort following neoadjuvant therapy

^a^Grade 1a = Complete histopathological response with no residual tumor cells

^b^Grade 1b = Subtotal response with minimal residual tumor cells

^c^Grade 2 = Partial response with significant residual tumor cells

^d^Grade 3 = Minimal or no response, indicating poor regression

The 21 patients included in the study were stratified into “Responder” and “Non-Responder” groups based on histopathological tumor regression following neoadjuvant therapy, as assessed in postoperative specimens using the Werner and Höfler grading system. The “Responder” group comprised 8 patients, whose tumors demonstrated either complete (Grade 1a) or subtotal (Grade 1b) regression. The remaining 13 patients were classified as “Non-Responders,” exhibiting limited therapeutic response, defined as partial (Grade 2) or minimal / no regression (Grade 3).

**Fig. 2:**
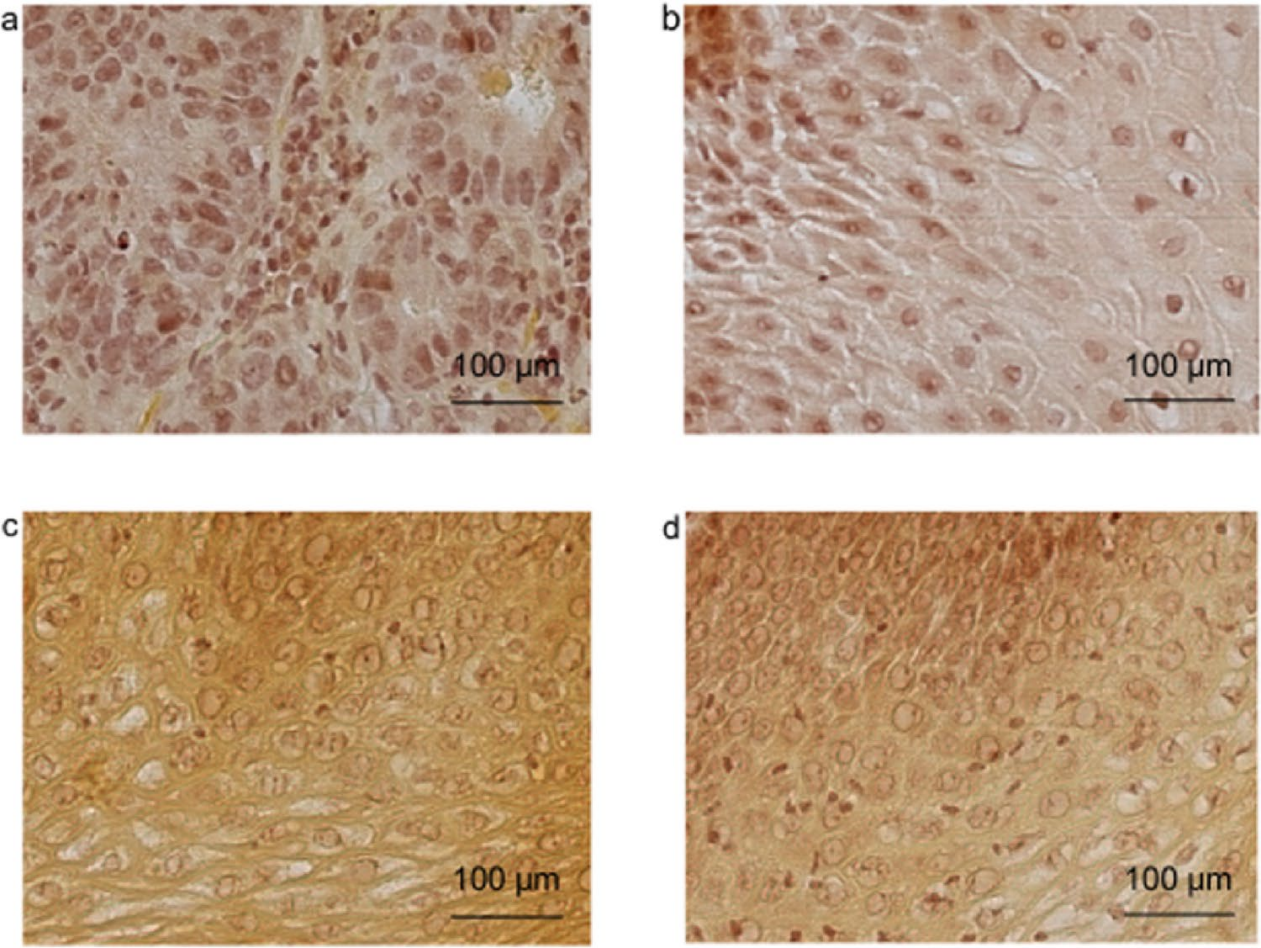
RGB images of an EAC responder to neoadjuvant therapy. Representative RGB images captured with the hyperspectral camera mounted on a microscope with a 20× objective, showing tissue specimens from a patient with a histopathological complete response (Grade 1a) according to Werner and Höfler. Images a and b display pretherapeutic HE-stained specimens, with image a showing EAC and image b showing healthy esophageal tissue. Images c and d display postoperative HE-stained specimens, where the pathological regression was defined as complete response by a specialized GI pathologist (KS). This patient was assigned to the “Responder” group (scale bar = 100 µm)

**Fig. 3:**
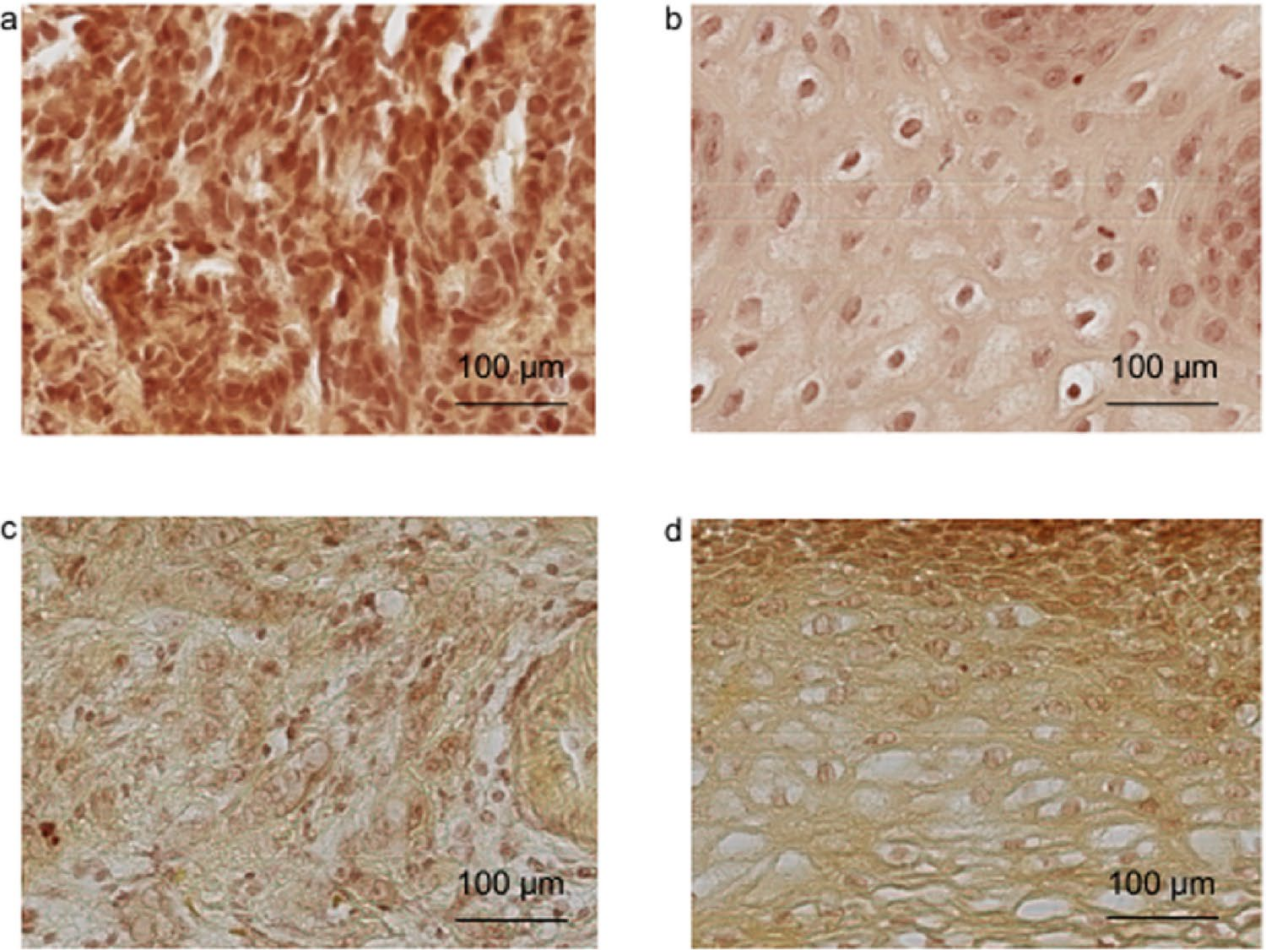
RGB images of a non-responder to neoadjuvant therapy. Representative RGB images captured with the hyperspectral camera mounted on a microscope with a 20× objective, showing tissue specimens from a patient with a histopathological response (Grade 2) according to Werner and Höfler. Images a and b display pre-therapeutic HE-stained specimens, with image a showing EAC and image b showing healthy esophageal tissue. Images c and d display postoperative HE-stained specimens, where the pathological regression grade was defined as partial response (Grade 2) by a specialized GI pathologist (KS). This patient was assigned to the “Non-Responder” group (scale bar = 100 µm)

To enable comparative assessment, tumor regression was additionally evaluated using the Schneider grading system. In this classification, Grade IV (no viable tumor cells) and Grade III (< 10% viable tumor cells) correspond to Grades 1a and 1b, respectively, in the Werner and Höfler system. Similarly, Grade II (10–50% viable tumor cells) and Grade I (> 50% viable tumor cells) align with Grades 2 and 3. The concordance between the two grading systems reinforces the validity and consistency of tumor regression assessment across different histopathological frameworks. Representative RGB (red, green blue) images acquired using the hyperspectral imaging system are presented in Figs. 2 and 3. Figure 2 displays pretherapeutic (a, b) and postoperative (c, d) tissue sections from a patient exhibiting a histopathological complete response, classified as a “Responder” based on the Werner and Höfler grading. In contrast, Fig. 3 depicts pretherapeutic (a, b) and postoperative (c, d) tissue sections from a patient with partial regression following neoadjuvant therapy, categorized as a “Non-Responder” according to the same classification.

### Classification

Three ANN architectures were evaluated to investigate their ability to predict EAC response to neoadjuvant therapy, based on HSI data from histopathological tissue specimens. The models included a 2D-CNN, a three- 3D-CNN, and a Hybrid-SN. Model performance was assessed using four standard evaluation metrics: accuracy, sensitivity, specificity, and F1-score (Table 3).

**Table 3:** Performance metric’s results

Model	Accuracy (%)	Sensitivity (%)	Specificity (%)	F1-Score (%)
2D-CNN	67 (± 12)	61 (± 14)	73 (± 15)	64 (± 15)
3D-CNN	68 (± 9)	68 (± 23)	68 (± 27)	66 (± 13)
Hybrid-SN	59 (± 18)	79 (± 19)	44 (± 48)	66 (± 8)

The table presents the accuracy, sensitivity, specificity, and F1-score for the three models (2D-CNN, 3D-CNN, and Hybrid-SN) used in the study. Values are reported as percentages with standard deviations (± SD) to indicate variability across the runs

For all models, the input patch size was set at 17 × 17 pixels, yielding a total of 4,608,000 data patches for training and evaluation. The 2D-CNN demonstrated moderate performance, primarily due to its strength in spatial feature extraction but limited capacity to capture spectral information. The 3D-CNN showed improved performance over the 2D-CNN by leveraging both spatial and spectral dimensions, resulting in enhanced predictive accuracy. Among the three, the Hybrid-SN model achieved the highest performance across all evaluation metrics. By combining 2D and 3D convolutional layers, it effectively integrated spatial–spectral features, making it the most robust and accurate model for EAC response prediction in this study.

### Visualization

**Fig. 4:**
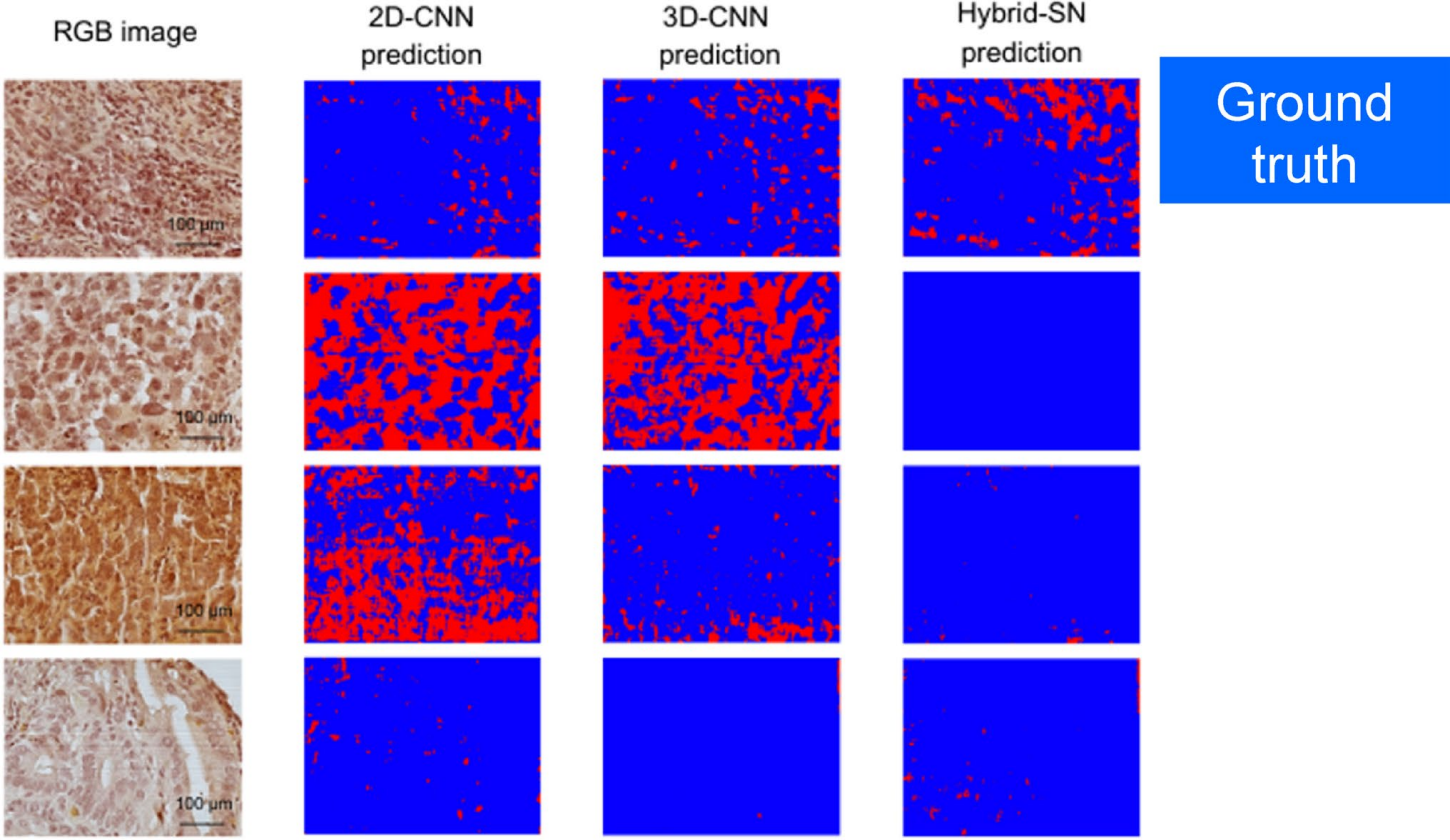
Prediction outputs for the „Responder “group. This figure displays the prediction outputs of the three neural network models (2D-CNN, 3D-CNN, and Hybrid-SN) for tissue samples from the „Responder “ group for four representative cases. The RGB images of the tissue samples are shown in the first column, followed by the prediction outputs of the 2D-CNN, 3D-CNN, and Hybrid-SN models in the subsequent columns. Blue regions represent „response “, which is the correct prediction for this group, as determined by the expert GI pathologist (KS). Mixed red and blue regions in the prediction outputs reflect the varying performance of the models and the spatial heterogeneity of the tissue samples (scale bar = 100 µm)

**Fig. 5:**
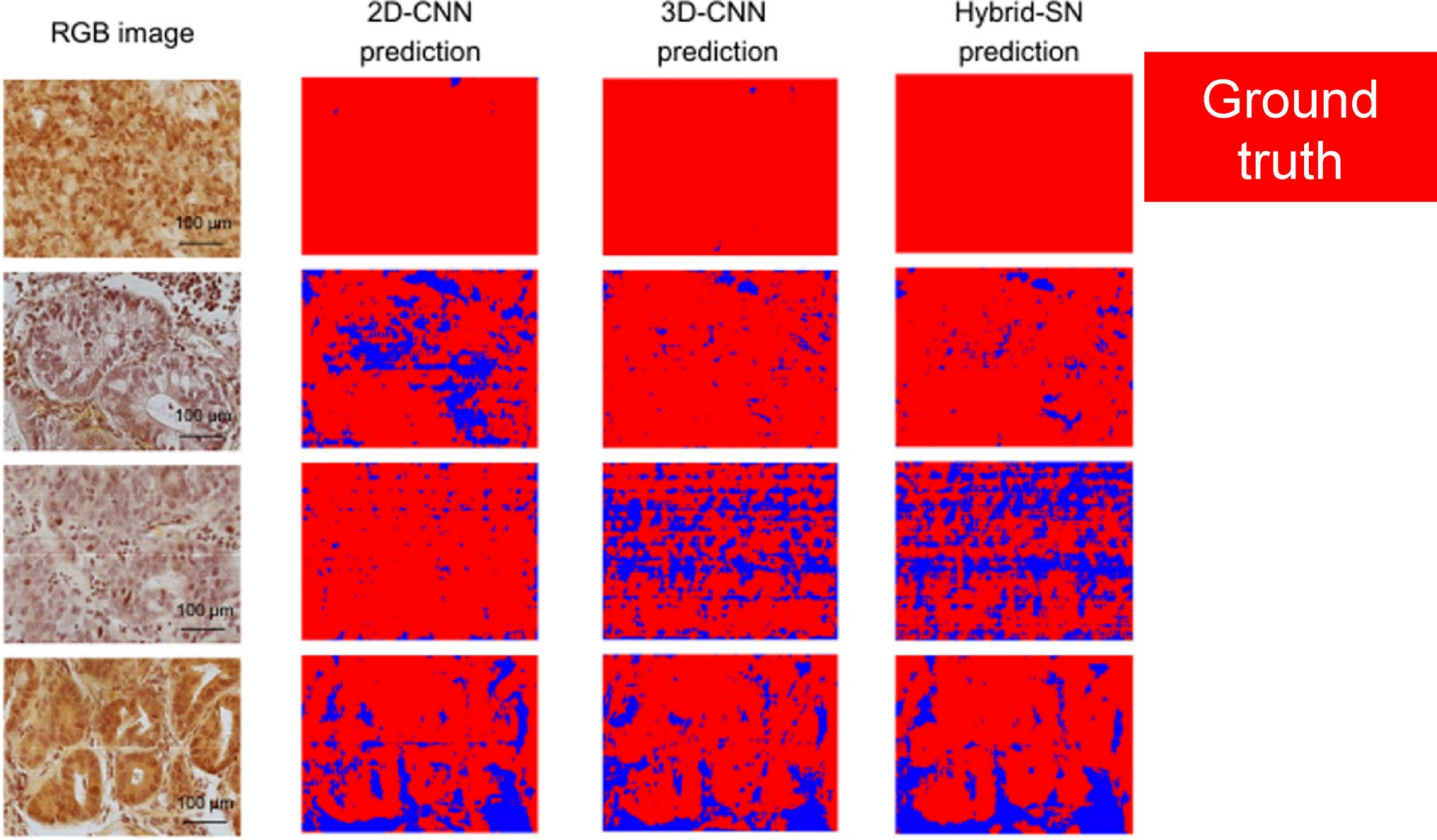
Prediction outputs for the „Non-Responder “ group. This figure displays the prediction outputs of the three neural network models (2D-CNN, 3D-CNN, and Hybrid-SN) for tissue samples from the “Non-Responder” group for four representative cases. The RGB images of the tissue samples are shown in the first column, followed by the prediction outputs of the 2D-CNN, 3D-CNN, and Hybrid-SN models in the subsequent columns. Red regions represent “no response,” which is the correct prediction for this group, as determined by the expert GI pathologist (KS). Mixed red and blue regions in the prediction outputs reflect the varying performance of the models and the spatial heterogeneity of the tissue samples (scale bar = 100 µm)

The classification outcomes generated by the three neural network models, 2D-CNN, 3D-CNN, and Hybrid-SN, for representative tissue samples from the “Responder” and “Non-Responder” groups are illustrated in Figs. 4 and 5. Predicted outputs are visualized as color-coded maps, with blue indicating a predicted “response” and red indicating “no response” to neoadjuvant therapy.

Figure 4 displays classification results for patients in the Responder group, where the expected outcome, based on histopathological evaluation, is “response” (blue regions). Conversely, Fig. 5 shows results for the Non-Responder group, where the correct classification is “no response” (red regions).

The classification maps reveal variability across the models, with some outputs exhibiting mixed blue and red regions, reflecting both model performance variability and underlying spatial heterogeneity within the tissue samples. Among the models, Hybrid-SN demonstrates superior spatial consistency and classification accuracy. Its predictions show the highest concordance with the expert-assigned histopathological regression grades, accurately identifying response in the “Responder” cases and lack of response in the “Non-Responder” cases.

The quantitative performance metrics of all three models, accuracy, sensitivity, specificity, and F1-score, are summarized in Table 4. These metrics further highlight the differential performance of each architecture, with the Hybrid-SN model consistently outperforming the 2D-CNN and the 3D-CNN models across all evaluation criteria.

2D-CNN Model:

- Achieved an accuracy of 67% (± 12%), correctly classifying approximately two-thirds of the samples. (Confidence interval (CI α = 0.05): 14%)
- Demonstrated the highest specificity (73% ± 15%), effectively identifying “Non-Responders”. (CI (α = 0.05): 17%)
- Sensitivity was moderate at 61% (± 14%), indicating limitations in identifying “Responders”. (CI (α = 0.05): 16%)
- F1-score was 64% (± 15%), slightly lower than the other models. (CI (α = 0.05): 17%)

3D-CNN Model:

- Achieved the highest accuracy (68% ± 9%) among the three models. (CI (α = 0.05): 10%)
- Sensitivity and specificity were balanced at 68% (± 23% and ± 27%, respectively), making it equally effective at identifying “Responders” and “Non-Responders”. (CI (α = 0.05): 26% and 29%, respectively)
- F1-score was 66% (± 13%), reflecting robust overall performance. (CI (α = 0.05): 15%)

Hybrid-SN Model:

- Achieved the highest sensitivity (79% ± 19%), excelling in identifying “Responders”. (CI (α = 0.05): 22%)
- Specificity was the lowest (44% ± 48%), indicating challenges in identifying “Non-Responders”. (CI (α = 0.05): 54%)
- Accuracy was 59% (± 18%), the lowest among the models. (CI (α = 0.05): 21%)
- F1-score was 66% (± 8%), comparable to the 3D-CNN model. (CI (α = 0.05): 9%)

## Discussion

Our findings support the hypothesis that HSI data can be effectively leveraged to predict the therapeutic response to neoadjuvant chemotherapy, chemoradiation, and immunotherapy in patients with EAC using ANNs. The evaluated models, 2D-CNN, 3D-CNN, and Hybrid-SN, demonstrated notable classification performance across key metrics, including accuracy, sensitivity, and specificity. Among the three architectures, the 3D-CNN achieved the highest overall accuracy and exhibited balanced performance across all evaluated metrics, indicating its robustness and versatility for EAC prognosis prediction. The Hybrid-SN model outperformed others in terms of sensitivity, highlighting its utility in scenarios, where the accurate identification of responders (true positives) is of primary importance. In this context, the Hybrid-SN is particularly well-suited for identifying patients likely to benefit from neoadjuvant therapy. The 2D-CNN model demonstrated the highest specificity, making it advantageous in applications, where minimizing false-positive classifications is critical. However, its relatively lower sensitivity may limit its utility in comprehensive predictive tasks. Each model’s distinct performance profile underscores the potential for tailored applications depending on specific clinical objectives—whether prioritizing sensitivity for treatment planning or specificity for reducing overtreatment. These results highlight the promise of ANN-based analysis of HSI data as a decision-support tool in personalized EAC treatment strategies.

Previous studies in cancer diagnostics have demonstrated the potential of integrating ANNs with HSI, yielding promising results. In the domain of prognosis prediction, Que et al. developed a preoperative ANN model to predict long-term survival in patients with gastric cancer, showing that the model outperformed the conventional TNM staging system in predictive accuracy. These findings align with the results of our study, which further support the utility of ANN-based approaches for predicting therapeutic outcomes in patients with EAC (Que et al. 2019). Collectively, this evidence highlights the growing role of ANN models as valuable tools in precision oncology. Jansen-Winkeln et al. demonstrated the potential of hyperspectral imaging (HSI) combined with neural networks in colorectal carcinoma, achieving high sensitivity (86%) and specificity (95%) for distinguishing cancerous from healthy mucosa (Jansen-Winkeln et al. 2019). For head and neck squamous cell carcinoma an improvement in classification accuracy to 82% was reported by combining HSI with CNN (Ma et al. 2022). These findings are consistent with the results of our study, in which the 3D-CNN model achieved balanced sensitivity and specificity, underlining the value of incorporating both spatial and spectral information in order to enhance classification accuracy in EAC. Variations in reported performance across studies may be attributable to differences in sample size, data pre-processing methods, and the architectural design of the neural networks employed. For instance, Courtenay et al. reported classification accuracies exceeding 90% for basal cell carcinoma using HSI and CNN, emphasizing the potential of these methods, but also highlighting the limitations in our own study related to sample size and dataset diversity (Courtenay et al. 2022). Another study by Ortega et al. combined HSI and deep learning for breast cancer detection, achieving an AUC of approximately 0.9 in differentiating tumor from normal tissue in histological samples (Ortega et al. 2020). Their approach included data augmentation strategies, such as spatial flipping and rotation, to address class imbalance and enhance model generalizability. Adopting similar augmentation techniques in future iterations of our study may help mitigate class imbalance between “Responder” and “Non-Responder” groups, reduce overfitting, and improve the robustness and clinical applicability of our HSI-based predictive models.

Several recent studies have investigated the integration of heterogeneous patient data—such as radiomics, genomics, pathomics, and clinical variables—into deep learning frameworks, demonstrating that the fusion of these complementary modalities can significantly enhance predictive accuracy and address key challenges in cancer diagnostics and therapy response prediction. Hörst et al. exemplified this approach by applying deep learning models, including multiple instance learning (MIL) and clustering-constrained attention MIL (CLAM), to predict therapy response in patients with gastroesophageal adenocarcinoma. Using pre-treatment and on-treatment PET/CT imaging alongside biopsy data, their study emphasized the utility of histologic biomarkers for enabling early therapeutic adjustments (Hörst et al. 2023). Importantly, the same study used pretrained models and attention heatmaps for interpretability, which provide insights that could enhance methodology, particularly in addressing tumor heterogeneity (Hörst et al. 2023). Their study further reported that therapy-induced tissue alterations increased intratumoral and peritumoral heterogeneity, which in turn influenced model performance. A similar phenomenon may have affected our hyperspectral imaging (HSI) results, as increased heterogeneity can introduce variability in spectral features and challenge the consistency of predictive outputs. Specifically, biochemical changes—such as variations in protein, lipid, and nucleic acid composition—can alter tissue spectral signatures. Concurrent structural alterations, including fibrosis, necrosis, and fluctuations in cellular density, influence light scattering and absorption properties. The presence of mixed histological regions containing viable tumor, necrotic tissue, and inflammatory infiltrates further complicates spectral interpretation. Addressing these sources of variability through model refinement, improved pre-processing, and potentially multimodal data integration will be critical in future studies to enhance the robustness and generalizability of HSI-based predictive models in oncology.

The potential of AI in predicting therapy response is further underlined by recent advancements in endoscopic evaluation for ESCC. In one study, twenty distinct AI models were trained to detect pathological complete response following neoadjuvant chemotherapy, achieving a mean AUC of 0.80, surpassing human endoscopists in terms of sensitivity. Despite these promising results, the approach was limited by relevant challenges, such as false-negative predictions, often caused by the presence of granulation tissue or suboptimal endoscopic focus (Matsuda et al. 2023). In contrast, our methodology, utilizing HSI of HE-stained histological specimens, offers enhanced diagnostic potential by capturing both spatial and spectral information. This dual-modality insight allows for more detailed tissue characterization, potentially overcoming the limitations observed in endoscopic-based AI models and improving the accuracy of therapy response prediction in esophageal cancer.

Recent advancements in multimodal data integration have shown considerable promise in enhancing the prediction of therapeutic response. Qi et al. developed a multimodality predictive model for assessing pathological complete response in esophageal cancer patients undergoing neoadjuvant chemoimmunotherapy. Their approach combined radiomics (from CT imaging), pathomics (from whole-slide histopathology images), and clinical features using Support Vector Machine (SVM) classifiers with a radial basis function kernel. By constructing both single-modality and fused multi-modality models, they demonstrated that the integrated model significantly outperformed individual modalities, achieving an AUC of 0.89, with high specificity (94.28%), but moderate sensitivity (66.7%). While this study reinforces the potential of SVM-based approaches, CNNs offer distinct advantages, particularly for image-based data (Qi et al. 2025). CNNs are capable of learning hierarchical features directly from raw inputs, enabling the detection of subtle morphological and biochemical patterns without the need for handcrafted feature extraction. In contrast, SVMs depend on predefined features and lack the capacity to automatically capture complex spatial or spectral hierarchies, potentially limiting their performance in high-dimensional imaging tasks. Furthermore, the integration of HSI into a multimodal predictive framework introduces several advantages. HSI is a non-invasive, radiation-free, and cost-effective modality that enables detailed spectral and spatial analysis of tissue, making it well-suited for longitudinal therapy monitoring. Unlike CT, HSI avoids ionizing radiation and may offer complementary biochemical insights. While this study focused on ex vivo analyses, the potential for in vivo HSI applications, such as real-time monitoring of tissue oxygenation, remains an exciting avenue for future research and clinical translation.

A study by Kawahara et al. explored the application of convolutional neural networks (CNNs) for predicting therapy response in ESCC using endoscopic imaging. By incorporating a range of image processing filters, including wavelet, Laplacian, and Sobel filters, they significantly enhanced feature extraction, achieving an AUC of 0.83 for predicting pathological complete response. Their approach focused primarily on macrostructural features derived from surface-level endoscopic images (Kawahara et al. 2022). In contrast, our study leverages HSI, which provides a richer dataset by capturing both spectral and spatial information at the microstructural level from histopathological tissue specimens. Nonetheless, the methodological strategies employed by Kawahara et al. offer valuable insights for optimizing HSI data analysis.

Another noteworthy application of CNNs in gastroesophageal cancers involves enhancing model interpretability and predictive performance by identifying key morphological features beyond simple staining intensity. In a recent study, a CNN-based approach achieved a balanced accuracy of 0.94 in predicting HER2-status in gastroesophageal adenocarcinoma using immunohistochemically stained tissue microarrays. This high level of accuracy was attributed, in part, to the model’s ability to recognize nuanced histological patterns relevant to HER2-expression (Pisula et al. 2023).

Hu et al. utilized a ResNet50-based deep learning framework (RN-SVM) to predict pathological complete response to neoadjuvant chemotherapy in ESCC using CT-imaging. Their model achieved an overall accuracy of 77.1%. A notable strength of their approach was the application of transfer learning: a ResNet50 model pre-trained on the ImageNet dataset was fine-tuned on their domain-specific imaging data. This strategy enabled effective feature extraction despite a limited sample size, while also reducing training time and computational demands (Hu et al. 2021). The possibility to use such pre-trained networks with our dataset is possible too. In Seidlitz et al. a U-Net with an efficientnet-b5 encoder pre-trained was used to classify hyperspectral images (Seidlitz et al. 2022). In future studies, this approach can be tested to improve the classifications.

Healthcare systems stand to benefit from the potential cost savings associated with reducing the need for invasive procedures and optimizing resource utilization through more accurate, non-invasive diagnostic tools. However, the findings of our study require validation in larger and more diverse cohorts to ensure population-level generalizability. The observed variability in specificity, particularly in the Hybrid-SN model, highlights challenges in accurately identifying true negatives, emphasizing the need for further model refinement and external validation before clinical implementation. Moreover, successful integration of these AI-driven tools into clinical workflows demands careful consideration of ethical and regulatory frameworks to ensure transparency, trust, and broad stakeholder acceptance (Racine et al. 2019). Key ethical concerns include obtaining informed patient consent, safeguarding data privacy, and addressing potential algorithmic bias (Harishbhai Tilala et al. 2024). These aspects must be rigorously addressed to support responsible deployment and equitable access to AI-enhanced diagnostic systems. Although the Hybrid-SN model demonstrates lower accuracy and specificity compared to alternative architectures, its higher sensitivity may offer advantages in clinical contexts, where identifying true positives is critical, such as early screening or high-risk triage. This reflects the inherent trade-off between sensitivity and specificity, underscoring the importance of selecting models based on clinical priorities rather than single performance metrics. Thus, while not universally “best,” the Hybrid-SN model may be preferable in settings where the cost of false negatives outweighs that of false positives. A robust model therefore need to show generalizability across populations, interpretability, computational efficiency, and resilience to data noise or missing data.

This study has several potential limitations. Selection bias may have been introduced due to restrictive inclusion criteria and the single-center design. We acknowledge that training and evaluating the models on a single-center dataset may limit generalizability. Single-center data can introduce sampling biases related to demographic, clinical practice, or equipment variability that may not reflect broader patient populations. While external validation is a critical next step, we emphasize that our findings serve as a proof-of-concept, and future work will focus on testing the models across diverse, multi-center cohorts to better assess their clinical applicability. Measurement bias is also a concern, particularly in the hyperspectral imaging (HSI) data acquisition process, where variable lighting conditions could have affected spectral consistency and image quality. It is also important to acknowledge the impact that staining of histopathological slides can have. Staining can influence model performance and limit the generalizability of trained models. Therefore, future research should consider the potential of analyzing pathological slides without staining. Additionally, the study highlights several research gaps. These include the limited sample size, the absence of large, comprehensively annotated datasets, variability in model evaluation metrics, and dependence on high-performance computing resources for training and inference. To address these limitations, future research should focus on the inclusion of larger, multi-center cohorts, the development of standardized, high-quality datasets, and the continued refinement of neural network architectures to enhance specificity, sensitivity, accuracy, and F1-score. These steps are essential to improve model robustness and facilitate clinical translation. Especially, the high standard deviation emphasize that further enhancements have to be done that the trained model achieved more homogeneous results.

ANNs leveraging HSI data hold substantial promise as prognostic tools in oncology. Future research should prioritize the expansion of datasets to encompass more diverse patient populations, thereby enhancing model generalizability and robustness. The integration of multimodal data, combining HSI with radiomics, pathomics, genomics, or proteomics, may offer a more comprehensive characterization of tumor biology and—furthermore—improve predictive accuracy. Establishing standardized protocols for hyperspectral data acquisition and ANN model training is crucial to ensure reproducibility and consistency across different clinical environments.

In future studies, the development of lightweight CNN architectures optimized for real-time processing should be done. These models offer the possibility to be incorporated to clinical workflows, enhancing their practicality for routine use. Gradual adoption of such technologies in real-time clinical settings, including during microscopic evaluation of histopathological specimens, has the potential to support pathologists’ diagnostic decisions and assist clinicians in prognosis prediction. This integration could accelerate traditionally time-intensive procedures and ultimately improve patient outcomes.

In conclusion, by addressing current limitations and capitalizing on emerging technological advances, ANN models based on HSI data can be refined to provide reliable, real-time decision support, advancing precision medicine and individualized patient care.

This study demonstrates that hyperspectral imaging data can be effectively utilized to predict therapeutic response in Barrett’s carcinoma using ANN-based models. We evaluated three neural network architectures—2D-CNN, 3D-CNN, and Hybrid-SN. The 3D-CNN model achieved the highest overall accuracy (0.68 ± 0.09) and F1-score (0.66 ± 0.08), reinforcing its strength in capturing both spatial and spectral features inherent to HSI data. The Hybrid-SN model exhibited superior sensitivity (0.79 ± 0.19), making it particularly effective in identifying patients likely to exhibit tumor regression following neoadjuvant therapy. These results highlight the potential of combining HSI and ANN techniques to enhance treatment response prediction in EAC patients, ultimately supporting the development of more personalized and effective therapeutic strategies. Nevertheless, challenges such as the need for larger, more diverse datasets, standardized acquisition and analysis protocols, and ethical considerations remain to be addressed before clinical implementation.

## Data Availability

The datasets generated during and/or analysed during the current study are available from the corresponding author on reasonable request.
